# Supplementary material for: Synthesis, Cytotoxicity Assessment and Optical Properties Characterization of Colloidal GdPO4:Mn2+, Eu3+ for High Sensitivity Luminescent Nanothermometers Operating in the Physiological Temperature Range
Source: Nanomaterials (Basel). 2020 Feb 28;10(3):421. doi: 10.3390/nano10030421 (PMC7152838; doi:10.3390/nano10030421)
Supplement: Supplementary file 1 [file nanomaterials-10-00421-s001.pdf]

Supplementary Information

Synthesis, Cytotoxicity Assessment and Optical Properties Characterization of Colloidal $GdPO_4:Mn^{2+}$, Eu^{3+} for High Sensitivity Luminescent Nanothermometers Operating in the Physiological Temperature Range

K. Maciejewska[1*], B. Poźniak[2], M. Tikhomirov[2], A. Kobylińska[1] and L. Marciniak[1*]

[1] Institute of Low Temperature and Structure Research, Polish Academy of Sciences, Okólna 2, 50-422 Wroclaw, Poland

[2] Wrocław University of Environmental and Life Sciences, ul. C.K. Norwida 31, 50-366 Wrocław

* Correspondence: k.maciejewska@intibs.pl; l.marciniak@intibs.pl

Table S1. The results of ICP measurments for selected nanocrystals.

Sample	% Mn	% Eu	% Gd
15% Mn	18.42113361	0	81.57887
1% Eu	0	1.312104	98.6879
1% Mn, 1% Eu	1.625904839	1.278901	97.09519
5% Mn, 1% Eu	6.324815405	1.20382	92.47136
10% Mn, 1% Eu	11.32800033	1.305311	87.36669
15% Mn, 1% Eu	17.80329943	1.313448	80.88325
15% Mn, 0.1%Eu	18.74500723	0.109384	81.14561
15% Mn, 0.2% Eu	17.76267241	0.219622	82.01771
15% Mn, 0.5% Eu	16.81513671	0.712126	82.47274

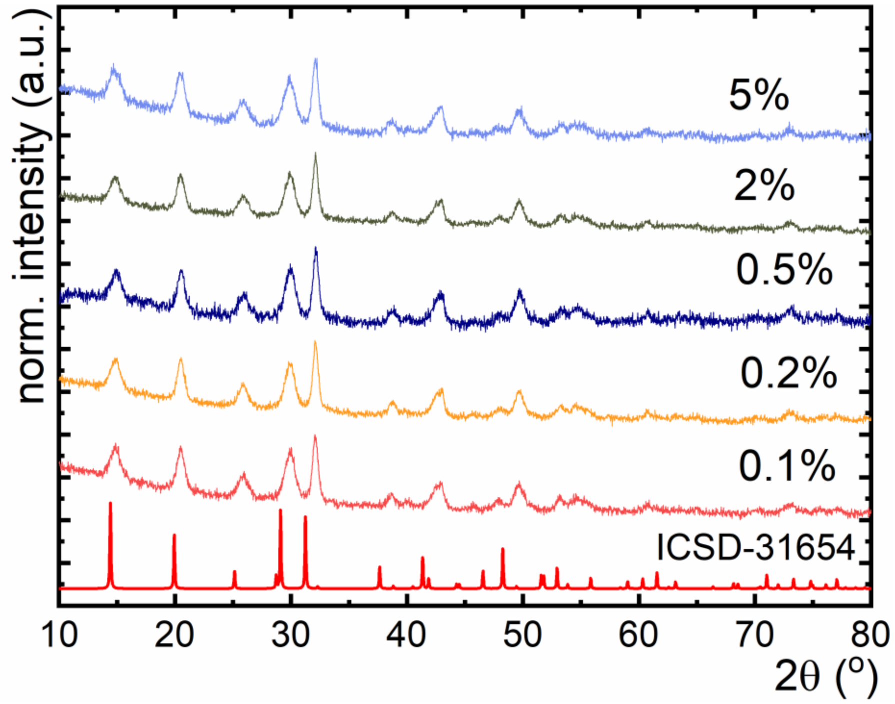

Figure S1. The comparison of X-ray diffractiograms of $GdPO_4$:$10Mn^{2+}$, x%Eu^{3+} with different concentration of Eu^{3+} ions.

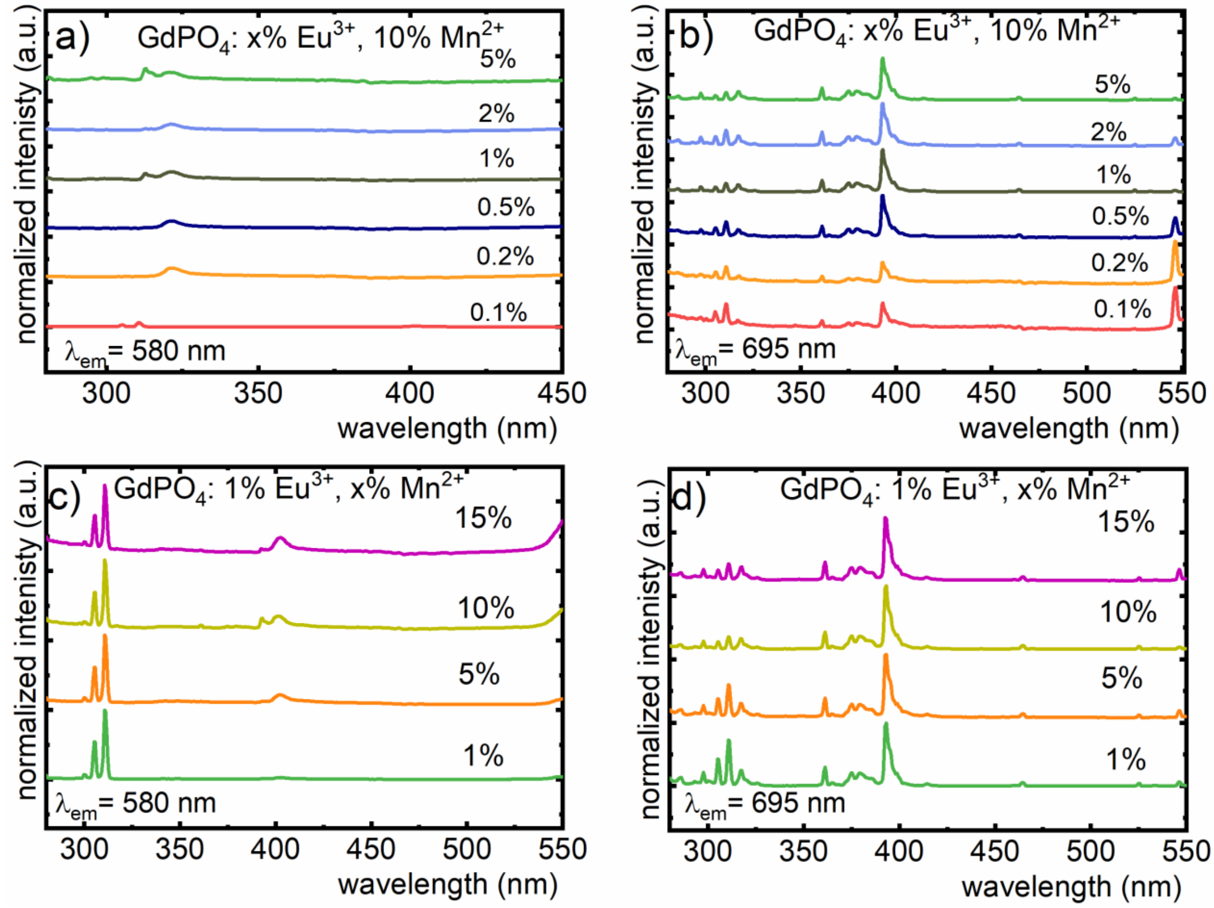

Figure S2. Excitation spectra of $GdPO_4$ doped with different concentration of Mn^{2+} (a, c) and Eu^{3+} (b, d) ions with emission monitored at 695 nm ($^5D_0 \rightarrow {}^7F_J$ electronic transition of Eu^{3+})- (c, d) and at emission 580 nm ($^4T_1 \rightarrow {}^6A_1$ electronic transition of Mn^{2+}) (a, b).

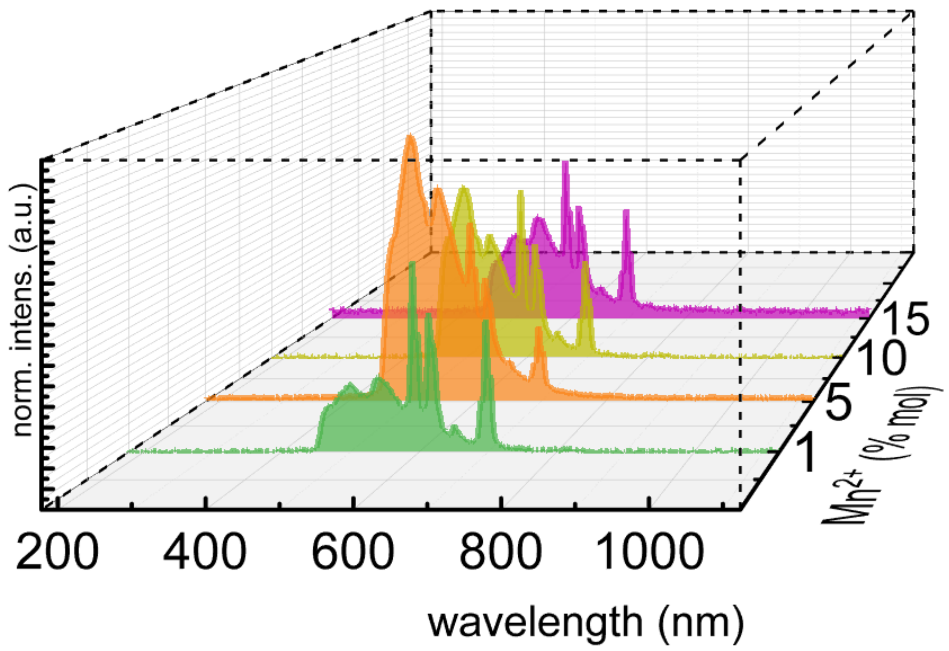

Figure S3. The comparisson of room temperature emission spectra of $GdPO_4$:1%Eu^{3+}, x%Mn^{2+} with different concentration of Mn^{2+} ions.

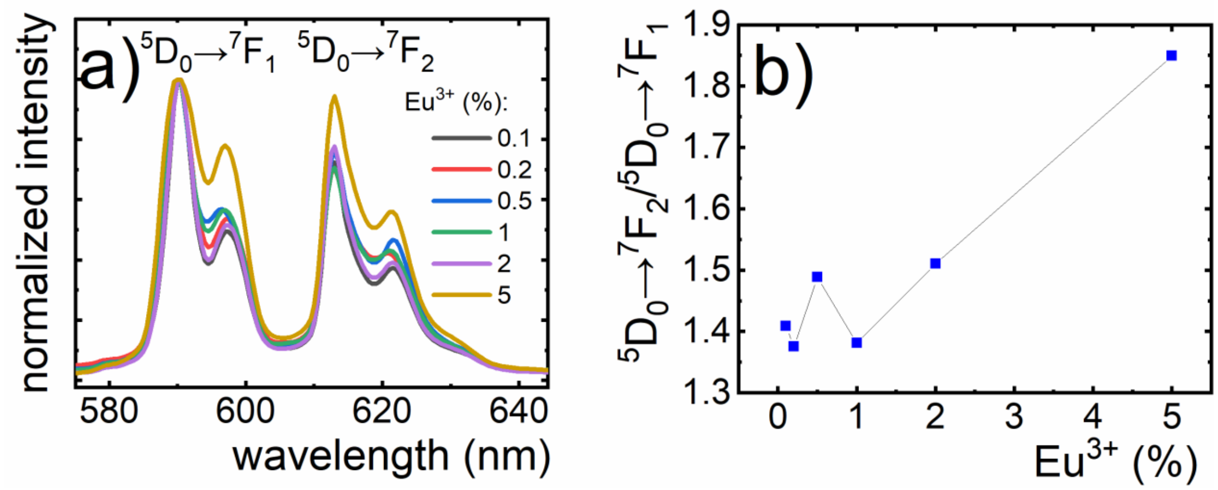

Figure S4. The comparisson of normalized (to the intensity of $^5D_0 \rightarrow {}^7F_1$ emission band) room temperature emission spectra of $GdPO_4$:Eu^{3+} nanocrystals with different concentration of Eu^{3+} ions – (a) and the disorder parameter (emission intensity ratio of $^5D_0 \rightarrow {}^7F_2$ band to the $^5D_0 \rightarrow {}^7F_1$ band)-(b).

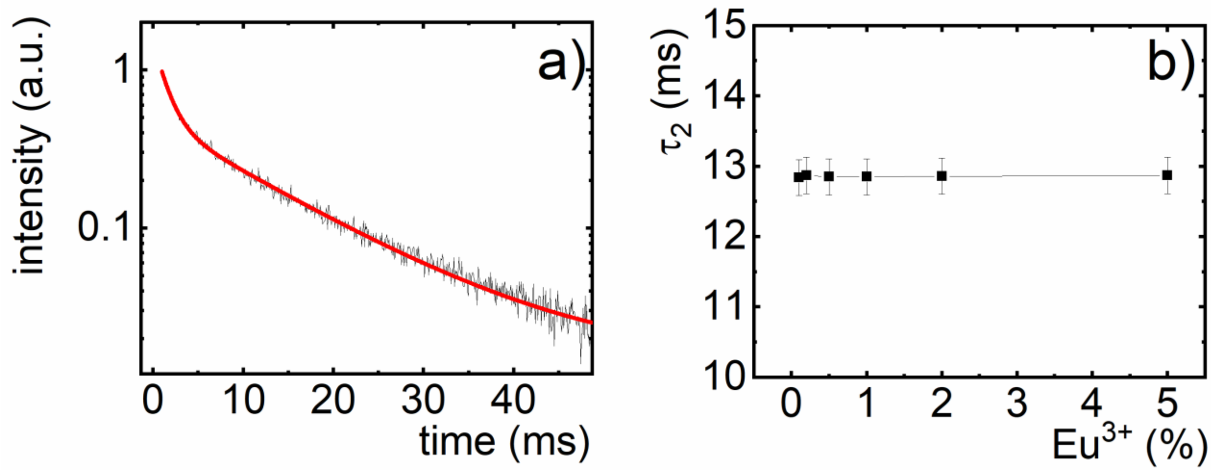

Figure S5. Luminescence decay profile of Mn^{2+} luminescence (λ_{em} = 550 nm) for $GdPO_4$:10%Mn^{2+}, 5%Eu^{3+} nanocrystals with double exponential fit-a) and lifetime τ_2 cslculated from double exponential fitting as a function of Eu^{3+} concentration-b).

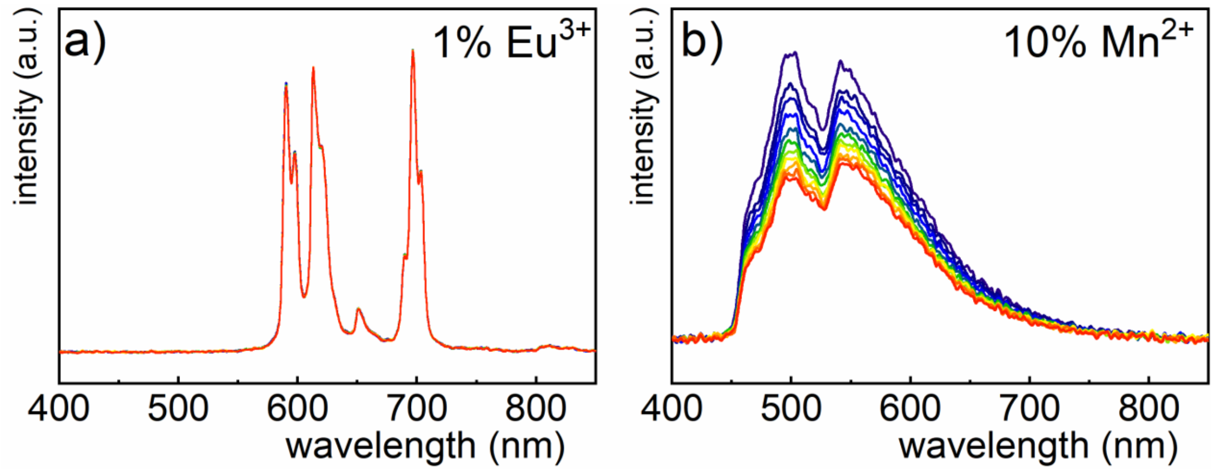

Figure 6. Thermal evolution spectra of $GdPO_4$ nanocrystals doped with: 1%Eu^{3+} (a) and 10%Mn^{2+} (b).

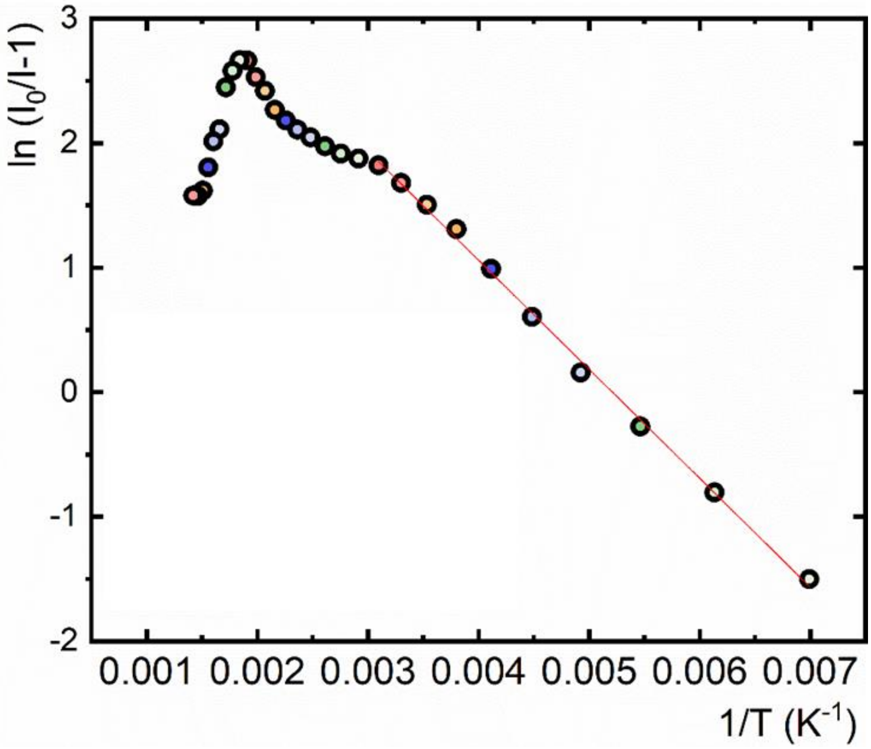

Figure S7. The ln(I0/I-1) vs 1/T plot for $GdPO_4$: 10% Mn^{2+} nanocrystals.

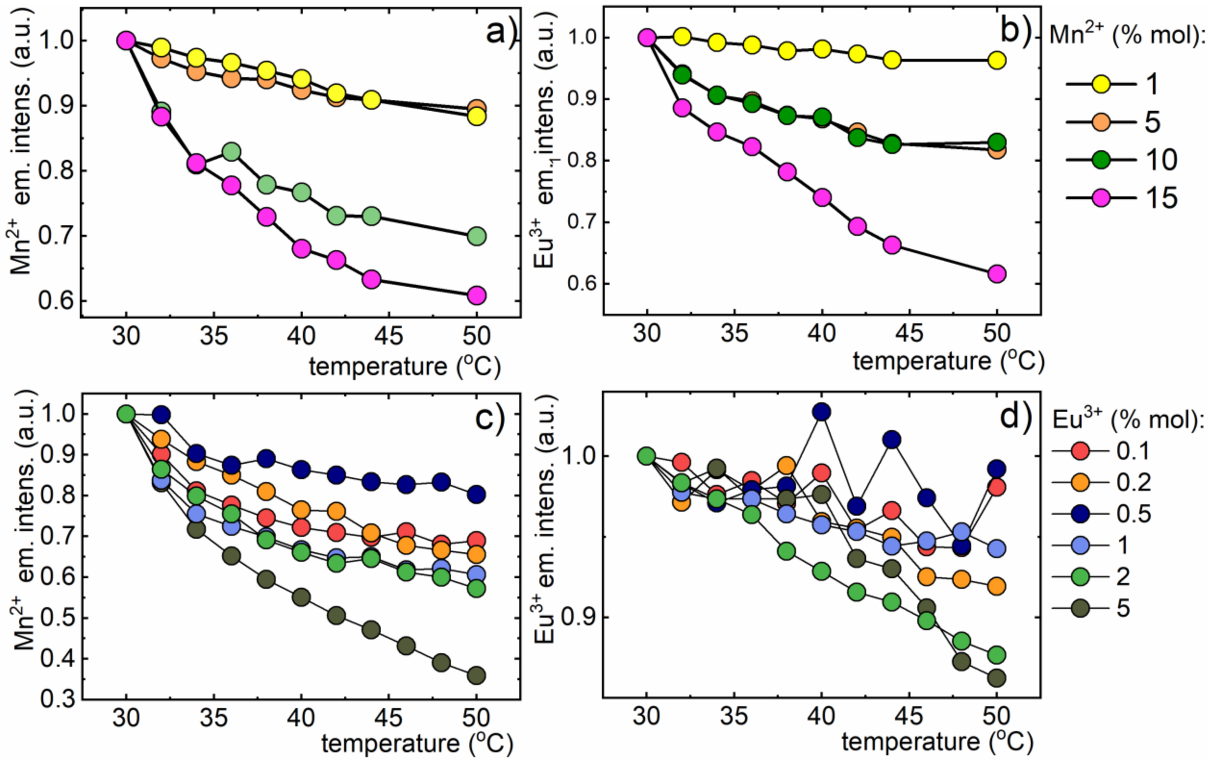

Figure S8. Thermal evolution of integrated Eu^{3+} (a, c) and Mn^{2+} (b, d) emission intensities for $GdPO_4$ doped with different concentration of Mn^{2+} (1, 5, 10, 15%) with 1% Eu^{3+} and Eu^{3+} (0.1, 0.2, 0.5, 1, 2, 5%) with 10% Mn^{2+}.

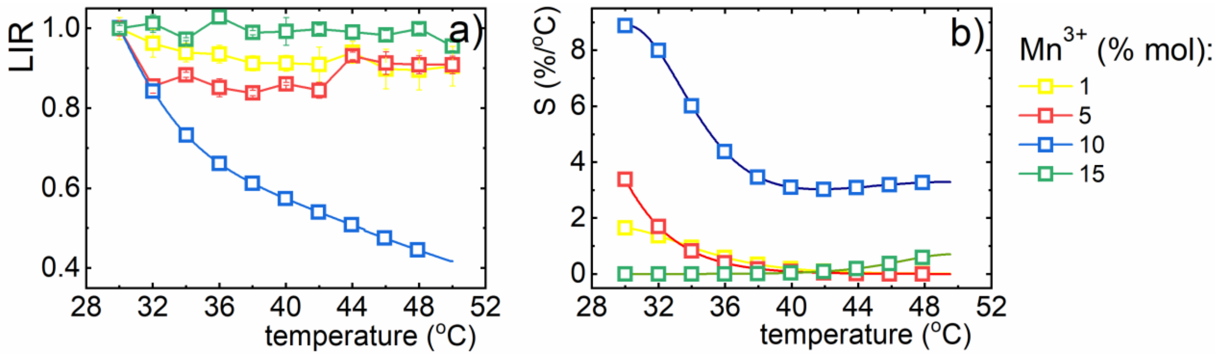

Figure S9. Thermal evolution of luminescence intensity ratio (LIR) (a) their relative sensitivities (b) for $GdPO_4$ doped with 1% Eu^{3+} and 1, 5, 10, 15 % Mn^{2+} nanoparticles.

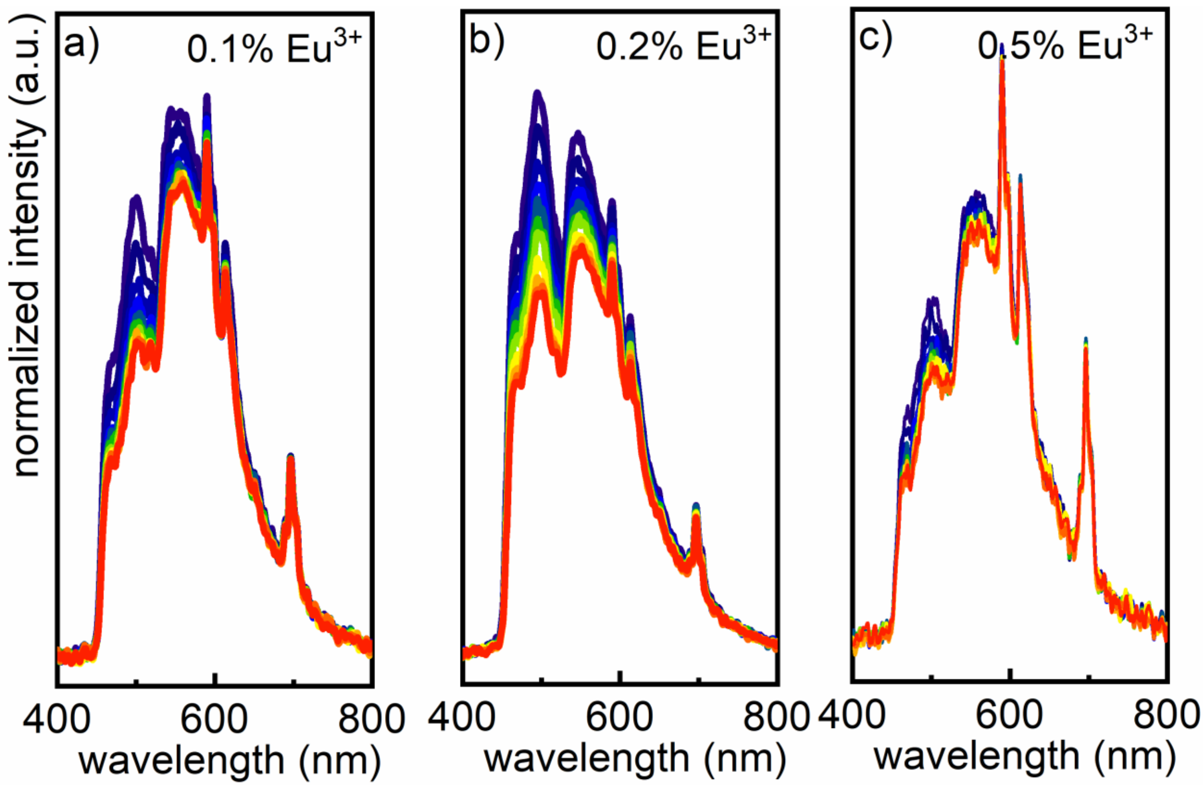

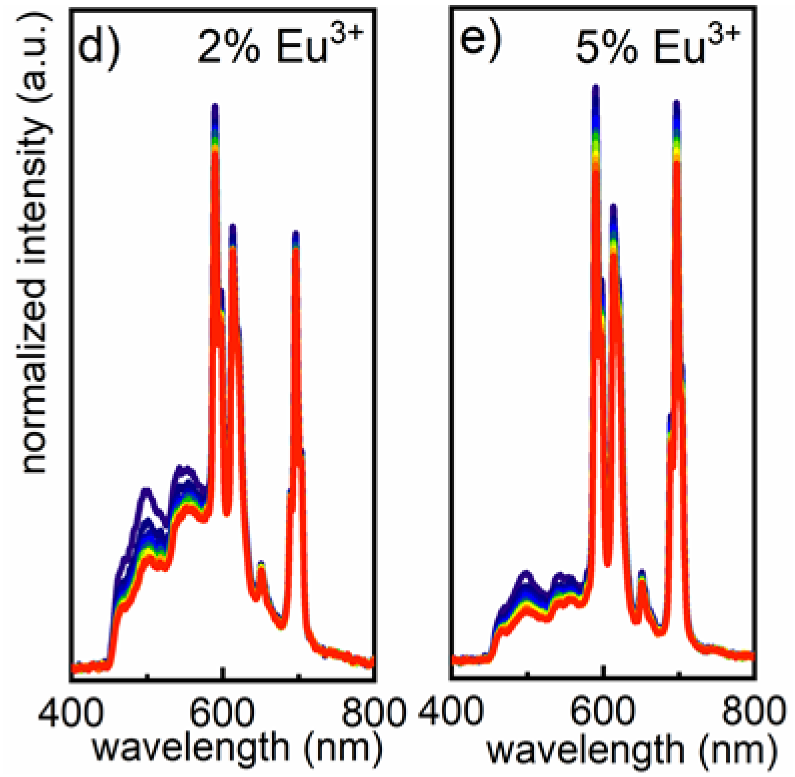

Figure S10. Thermal evolution spectra of $GdPO_4$ nanocrystals doped with: 0.1%Eu^{3+}, 10%Mn^{2+}-(a); 0.2%Eu^{3+}, 10%Mn^{2+}-(b); 0.5%Eu^{3+}, 10%Mn^{2+}-(c); 2%Eu^{3+}, 10%Mn^{2+}-(d); 5%Eu^{3+}, 10%Mn^{2+}-(e) monitored at 30-50 ^{o}C.

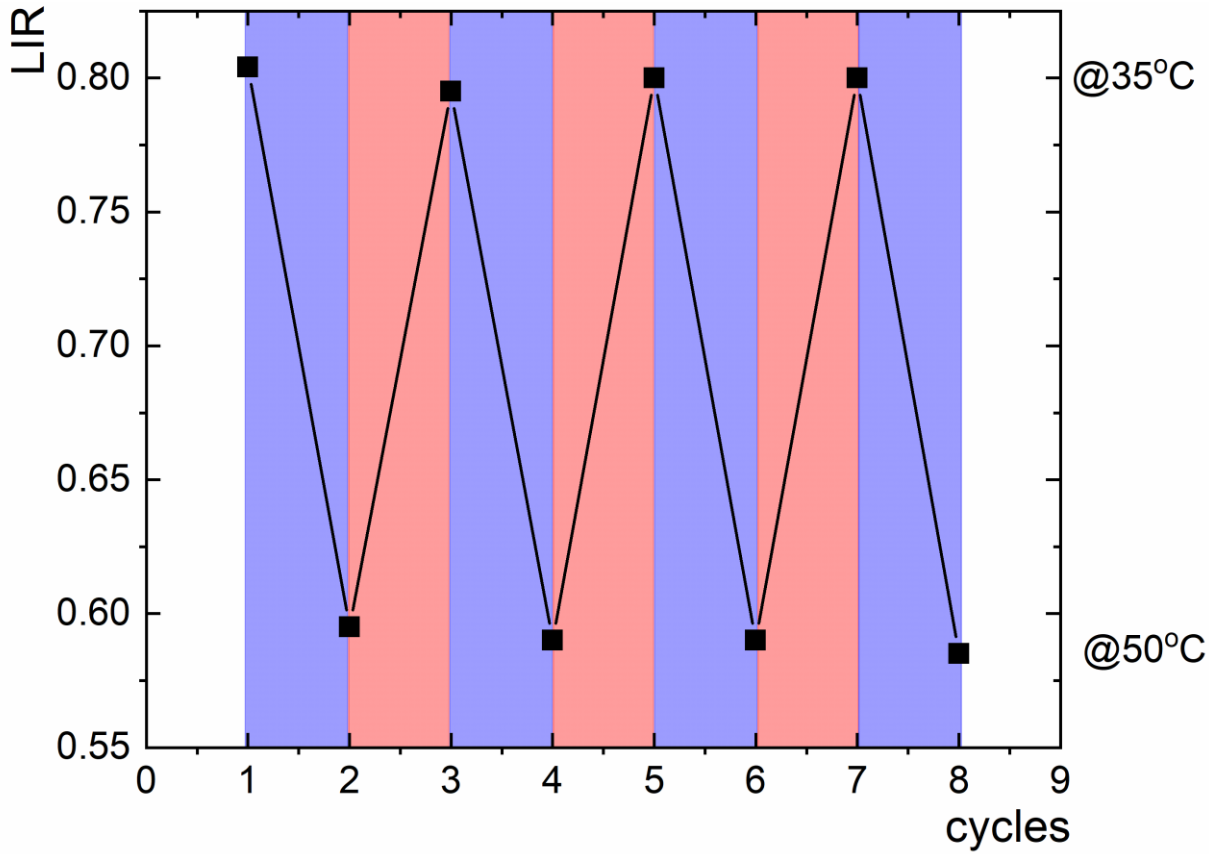

Figure S11. Thermal dependence of LIR for the $GdPO_4$ 10% Mn^{2+}, 1%Eu^{3+} nanocrystals during heating-cooling cycles.
